# Does omission of a loose seton before sphincter-preserving surgery achieve non-inferior fistula closure compared with routine loose seton placement in adults with single-tract cryptoglandular anal fistula in the Netherlands? Study protocol for the SETON multicentre randomised controlled non-inferiority trial and cost-utility analysis

**DOI:** 10.1136/bmjopen-2026-122832

**Published:** 2026-10-06

**Authors:** Ahmed A Elwakel, Pieter Van Hagen, Ruben Veldkamp, Susan van Dieren, Willem A Bemelman, Ingrid J M Han-Geurts, The SETON projectgroup, I.J.M. Han-Geurts

**Affiliations:** 1 Department of Surgery, Amsterdam UMC, Amsterdam, The Netherlands; 2 Department of Surgery, Proctos Kliniek, Bilthoven, The Netherlands

**Keywords:** Colorectal surgery, SURGERY, Adult surgery

## Abstract

**Abstract:**

**Introduction:**

Cryptoglandular anal fistulas are burdensome, often recurrent and frequently treated in two stages using a loose seton before definitive sphincter-preserving surgery. Although this practice is common, high-quality evidence supporting improved healing or functional outcomes is lacking. A trial is needed to determine whether omitting routine seton placement can reduce procedures, patient burden and costs without compromising fistula closure.

**Methods and analysis:**

The SETON trial is a multicentre, randomised, unblinded non-inferiority trial with an integrated cost-utility analysis. Adults with a single-tract non-purulent mid-high or high cryptoglandular anal fistula will be randomised to loose seton placement for 8 weeks followed by definitive sphincter-preserving surgery, or to direct sphincter-preserving surgery without prior seton placement. The primary endpoint is fistula closure 26 weeks after final surgery, defined clinically and if needed confirmed by MRI or endoanal ultrasound. Secondary outcomes include recurrence, postoperative complications, pain, re-interventions, continence, fistula-related quality of life, generic health-related quality of life, return to work and cost-effectiveness. A sample size has been calculated for a non-inferiority design with fistula closure at 26 weeks as the primary endpoint. Assuming a conservative closure rate of 60% in the standard-care seton group, a one-sided alpha of 0.05, 80% power and a non-inferiority margin of 15 percentage points, 126 patients are required per group for analysis. Allowing for approximately 10% loss to follow-up, the total sample size will be 277 patients. Data will be analysed according to the intention-to-treat and per-protocol principle.

**Ethics and dissemination:**

The protocol has been approved by the Medical Ethics Review Committee of the Amsterdam University Medical Center, location AMC (reference number: 2025.0826—NL-010338 R031541). Findings will be disseminated in peer-reviewed journals and presented at conferences, whether they are positive, negative or inconclusive.

**Trial registration number:**

NL-010338

**STRENGTHS AND LIMITATIONS OF THIS STUDY**

- Pragmatic multicentre randomised design comparing two established treatment pathways used in current clinical practice.
- Evaluates both clinical outcomes and patient-centred outcomes, including continence, quality of life and return to work.
- Includes an economic evaluation from a societal perspective to inform efficient allocation of care.
- Blinding is not feasible because the compared strategies are visibly different surgical pathways.

## Introduction and rationale

 Perianal fistulas are a burdensome and often recurrent condition that can substantially affect quality of life and lead to prolonged treatment trajectories. In the Netherlands, approximately 5000 surgical interventions are performed annually for this condition, and more than half of patients undergo two or more procedures.^1^ Mid-high and high cryptoglandular fistulas are often treated by a staged strategy beginning with placement of a loose seton before definitive repair.^1^

Although loose seton placement is considered standard practice in many centres, evidence supporting superiority over direct surgery is weak. The European Society of Coloproctology guideline acknowledges the practice but rates the supporting evidence as low, and the Dutch proctology guideline does not provide a strong recommendation for routine preoperative seton use.^1 2^

A seton may theoretically promote drainage, reduce inflammation and permit tract maturation before definitive surgery, but it also adds an extra procedure, extends treatment duration and may increase inconvenience for patients. Retrospective data suggest that preoperative seton drainage does not clearly improve outcomes before definitive repair.^3^ From both clinical and health-economic perspectives, it is therefore relevant to determine whether this routine step is necessary.

The primary objective of this trial is to determine whether direct sphincter-preserving surgery without prior loose seton placement is non-inferior to the current two-stage strategy with loose seton placement for fistula closure at 26 weeks after final surgery. Secondary objectives are to compare recurrence, postoperative complications, re-interventions, pain, continence, fistula-related and generic quality of life, return to work, healthcare costs and cost-utility between the two treatment strategies.

### Hypothesis

Omitting routine loose seton placement before definitive sphincter-preserving surgery is non-inferior to the two-stage strategy for fistula closure at 26 weeks, while reducing treatment burden and improving cost-effectiveness.

## Methods and analysis

### Study design

The SETON trial is a multicentre, parallel-group, randomised controlled non-inferiority trial with an integrated cost-utility analysis (CUA). This trial was registered at the Dutch Trial Registry (NL-010338) prior to the start of inclusion. Consecutive eligible adults with a single-tract non-purulent mid-high or high cryptoglandular anal fistula will be recruited at participating Dutch centres and randomised in a 1:1 ratio. The unit of randomisation is the individual participant. Because the compared strategies differ visibly, the trial is unblinded for participants and treating surgeons. Primary non-inferiority analyses will be performed on both the per-protocol and intention-to-treat populations, with the per-protocol analysis leading to the non-inferiority conclusion.

### Eligibility criteria

#### Inclusion criteria

Age 18 years or older.

Single-tract mid-high or high cryptoglandular anal fistula, established on clinical grounds and confirmed by endoanal ultrasound or MRI when required.

Able to complete Dutch-language questionnaires sent by email.

### Exclusion criteria

Anorectal abscess.

Multiple or complex anorectal fistulas.

Inflammatory bowel disease or clinical suspicion requiring further investigation.

History of anorectal malignancy.

Use of immunosuppressive medication at the time of surgery.

History of pelvic radiation.

Pregnancy or lactation.

### Interventions

The definitive sphincter-preserving procedure will be selected before randomisation by the treating surgeon on the basis of fistula anatomy, including tract height and configuration and according to procedures routinely performed at the participating centre. The planned procedure will be documented before treatment allocation and used as a stratification factor. Only sphincter-preserving techniques that are part of standard practice within the participating centres, including mucosal advancement flap, ligation of the intersphincteric fistula tract and laser ablation of the fistula tract, are permitted within the trial.

### Loose seton strategy

Participants allocated to the intervention arm will undergo outpatient loose seton placement. A silicone vessel loop will be placed through the fistula tract as a non-cutting seton. After approximately 8 weeks, definitive sphincter-preserving surgery will be performed.

### Direct surgery strategy

In the direct surgery group, no preoperative loose seton will be placed as part of the trial strategy. Participants will proceed directly to the planned definitive sphincter-preserving procedure according to local scheduling and standard perioperative care. In participating centres, both a staged strategy with loose seton drainage followed by definitive sphincter-preserving surgery and a one-stage strategy with direct sphincter-preserving surgery are used in routine care. The trial compares these existing care pathways without introducing experimental procedures or trial-specific modifications to the surgical techniques themselves. All procedures will be performed or supervised by surgeons experienced in anal fistula surgery at the participating centres.

### Concomitant care and protocol adherence

No trial-specific co-interventions or escape medications are planned. Relevant concomitant care will be recorded. Adherence to the allocated strategy will be assessed from operative records, including whether participants received the assigned treatment pathway and reasons for protocol deviations or crossover.

### Outcomes

The primary outcome is successful fistula closure at 26 weeks after final surgery, defined as absence of an external opening or discharge on physical examination and confirmed by MRI or endoanal ultrasound if clinical uncertainty remains. Secondary outcomes include postoperative complications, need for re-intervention, pain intensity, time to return to work, continence assessed with the Vaizey score, fistula-related quality of life assessed with the Fecal Incontinence Quality of Life (FIQL), generic quality of life assessed with the EQ-5D-5L, patient-reported anorectal symptoms measured with Proctology Patient-Reported Outcome Measurement (proctoPROM)^4^ and healthcare and societal costs (table 1). Outcomes are reported in line with the Anal Fistula Core Outcome Set.

**Table 1:** Trial scheme with planned participant-reported assessments. iPCQ, iMTA Productivity Cost Questionnaire; iMCQ, iMTA Medical Consumption Questionnaire.

Measure	Baseline	1 week	6 weeks	6 months	12 months
EQ-5D-5L	x	x	x	x	x
proctoPROM	x	x	x	x	x
Vaizey/FIQL	x		x	x	x
iPCQ			x	x	x
iMCQ			x	x	x

### Participant timeline

### Sample size calculation

The sample size calculation is based on a non-inferiority comparison for fistula closure. The expected healing rate in the standard-care group was set at 60%.^5 6^ Using a non-inferiority margin of 15 percentage points, 80% power and a one-sided 5% significance level, 126 participants per group are required for analysis. Allowing for approximately 10% loss to follow-up, the total required sample size is 277 participants.

### Randomisation and allocation concealment

After fully signed written informed consent and collection of baseline data, participants will be randomly allocated in a 1:1 ratio through the secure web-based Castor Electronic Data Capture (EDC) system. The randomisation sequence will be computer generated. Randomisation will be stratified by participating centre and by planned sphincter-preserving treatment method to achieve a balanced distribution between treatment arms. Recruiters and treating teams will not be able to access the randomisation sequence before assignment. The specific details of any blocking or other allocation restrictions will be maintained outside the operational protocol to minimise predictability.

### Patient and public involvement

The patient organisation Bekken4all was consulted during the initial development of the study proposal to ensure that the study addressed issues relevant from the patient perspective. The organisation remained actively involved in the further development of the study protocol. Attention was given to the burden of trial participation and to patient-related outcomes. In addition, the patient organisation contributed to the preparation of patient information materials. Participants were provided with the contact details of the patient organisation in case they had questions.

### Recruitment and retention

Potentially eligible patients will be identified during outpatient clinic assessments at participating centres. Treating surgeons or delegated trained research staff will introduce the study, provide written information and allow at least 48 hours for consideration before consent. Reminder procedures by email or telephone will be used to improve questionnaire completion and follow-up. Reasons for withdrawal, non-adherence and loss to follow-up will be documented.

### Data collection and management

Data will be collected in Castor EDC using coded participant identifiers that are not derived from initials or dates of birth. Local investigators will maintain the code key in the investigator site file. Questionnaire data will be collected electronically. Access to the data will be restricted to authorised investigators, study staff, monitors and quality assurance personnel. All adverse events that were spontaneously reported by the participant or observed by the investigator or study staff will be recorded. Data will be retained for 15 years in accordance with applicable Dutch and European requirements.

### Statistical analysis

Data will be analysed according to both the intention-to-treat and the per-protocol principle. No interim analysis is planned. Analyses will be performed using SPSS V.26.0. The primary outcome, fistula closure, will be analysed by comparing the difference in healing proportions between the two groups, together with the corresponding two-sided 90% CI, consistent with a one-sided 5% non-inferiority test. Non-inferiority will be considered demonstrated if the lower bound of the CI lies above the prespecified non-inferiority margin of −15 percentage points. Secondary outcomes will be analysed using either a t-test or Mann-Whitney U test for continuous data and a χ^2^ test or Fisher’s exact test for categorical data. Questionnaire outcomes measured at repeated time points will be analysed using linear mixed models. Some missing data can be expected; if more than 10% of data are missing and these are judged to be missing at random, this will be handled using multiple imputations with predictive mean matching.

### Cost-effectiveness analysis

#### General considerations

This study will also include a full economic evaluation comparing the two treatment strategies. We hypothesise that omission of routine seton placement may reduce overall healthcare costs without compromising outcomes. The economic evaluation will be performed as both a cost-effectiveness analysis (CEA) and a CUA. In the CEA, the primary outcome will be cost per successfully healed fistula. In the CUA, the outcome will be cost per quality-adjusted life year (QALY). These analyses will help determine whether avoiding the seton step is not only clinically safe, but also a more efficient use of healthcare resources.

Both evaluations will be conducted from a societal perspective over a 12-month time horizon. In addition, a lifelong simulation will be performed using Monte Carlo methods to explore long-term cost differences. To address uncertainty, a probabilistic sensitivity analysis will be performed. Incremental cost-effectiveness ratios will be calculated for both healed fistulas and QALYs. Non-parametric bootstrapping will be used to assess variability and 95% CIs will be reported. The results will be presented using cost-effectiveness planes and acceptability curves. Sensitivity analyses will be performed to explore the influence of key cost assumptions

### Cost analysis

A cost analysis will be carried out from both the hospital and from the societal perspectives. Medical costs, patient costs and productivity losses will be included in the evaluation, in accordance with the most recent guidelines for costing in healthcare research.^7^ Medical costs will comprise direct healthcare costs, including surgery, hospital stay, follow-up visits and treatment of complications. Patient costs will include out-of-pocket expenses, such as travel costs and over-the-counter medication. Productivity losses will reflect absence from work.

Hospital healthcare utilisation will be obtained from patient records and case report forms. Postdischarge healthcare use will be assessed using the iMTA Medical Consumption Questionnaire, and productivity losses will be measured using the iMTA Productivity Cost Questionnaire. Questions on out-of-pocket expenses will be incorporated into these patient questionnaires. Patients will complete the questionnaires at baseline and at 3, 6 and 12 months.

Costs will be price-indexed using Consumer Price Indices and calculated for each individual patient as the product sum of resource use and the corresponding unit costs. In the lifelong evaluation, discounting of costs and effects will be applied to account for time preferences.

### Patient outcome analysis

To measure the effect on quality of life, all patients will complete the EQ-5D-5L questionnaire. These scores will be converted into utility values using the Dutch tariff^8^ and used to calculate QALYs through linear interpolation over time. The primary clinical outcome—fistula closure—will be recorded from the hospital data.

### Budget impact analysis

#### General considerations

The affordability of omitting routine seton placement in the treatment of high cryptoglandular perianal fistulas will be assessed from both a societal and hospital perspective, following the International Society for Pharmacoeconomics and Outcomes Research(ISPOR) guidelines for budget impact analysis.^9 10^ From a societal standpoint, this evaluation explores whether skipping the seton—if shown to be non-inferior—could lead to better resource allocation in the Dutch healthcare system. From the hospital perspective, the focus is on how this shift might affect actual operating budgets. The analysis will use a 5-year time horizon and will be both incidence- and prevalence-based, reflecting the impact of this change over time. Different implementation scenarios will be modelled: full immediate adoption, phased adoption (eg, a 25% increase in uptake each year), or partial uptake (eg, 70%, 80% or 90% coverage). Sensitivity analyses will explore the influence of variation in patient numbers, cost estimates and seton usage rates.

### Cost analysis

In the Netherlands, about 5000 anal fistula surgeries are performed annually.^11^ As precise usage rates of seton placement vary and are not consistently documented in national data, we are conservatively assuming that every one of these patients currently receives a seton. That means approximately 5000 patients per year would be impacted by this change in practice.

Dutch hospital tariffs and Diagnosis Treatment Combination (DBC) codes estimate the total cost of placing and managing a seton at around €1,000 per patient. This total incorporates the minor procedure (typically €450–€550), materials (€50–€100), one or more follow-up visits (€150–€200) and seton replacements or adjustments in about a quarter of cases (€100–€200).^11^

This translates to around €5 million in annual healthcare costs related to seton use. Beyond this, indirect societal costs also need to be considered. Waiting for definitive surgery due to the seton step may delay recovery and return to work. If patients in a one-stage strategy return to work just 5 days earlier on average—and applying a Dutch daily productivity loss of €278^7^—this could save €1390 per patient in lost productivity. Across the 5000 patients, that is an additional €7 million in potential societal savings per year. We expect to avoid a routine seton placement in 500 patients. That would mean a saving of €1 620 000 annually.

### Other study parameters

Baseline characteristics will be collected and summarised using descriptive statistics, including numbers and percentages for categorical variables and means with SD or medians with quartiles, as appropriate, for continuous variables. Differences between groups will be analysed using the independent Student’s t-test for normally distributed continuous data, the Mann-Whitney U test for non-normally distributed continuous data and the χ² test for categorical data.

### Ethics and dissemination

#### Regulation statement

The study will be conducted in accordance with the principles of the Declaration of Helsinki, the Medical Research Involving Human Subjects Act (Dutch WMO), and other applicable European guidelines, regulations and legislation, including the General Data Protection Regulation (in Dutch: Uitvoeringswet AVG). The protocol was approved by the Medical Ethics Review Committee of Amsterdam University Medical Centers, location Academic Medical Centre (reference number: 2025.0826—NL-010338 R031541). Study findings will be disseminated through peer-reviewed publications and conference presentations, irrespective of whether the results are positive, negative or inconclusive.

### Consent and confidentiality

Informed consent will be obtained by the treating physician or by a trained delegated member of the research team who is familiar with the trial procedures, risks and potential benefits. Preferably, informed consent will be obtained by the treating physician. Patients will receive oral and written information explaining that the study involves research, the procedures to be followed, the nature, expected duration and purpose of the study, and the potential risks, advantages and disadvantages of participation. They will also be informed about any reasonably expected benefits. The participant information sheet and informed consent form are provided as online supplemental material 1.

Participation will be voluntary, and declining participation will not affect usual care. Patients will be given sufficient time to consider participation and to ask questions before making a decision. Confidentiality will be protected through coded data handling and restricted access to identifiable information, and study data will be stored for 15 years. Any optional future-use consent statements will be aligned specifically with anal fistula research before publication.

### Compensation for injury

The sponsor/investigator has liability insurance in accordance with Article 7 of the WMO and the applicable legal requirements in the Netherlands. This insurance provides coverage for damage suffered by research participants because of injury or death caused by the study. The coverage applies to damage that becomes apparent during the study or within 4 years after the end of the study.

### Methods of dissemination of results

The study findings will first be discussed at a meeting of the Dutch Society of Coloproctology/Surgery. During this meeting, experiences from surgeons involved in the treatment of anal fistulas will be considered, the implications of the trial results will be outlined and practical aspects of implementation will be discussed. This will be followed by presentation of the results at national scientific meetings, including the annual meeting of the Dutch Society of Surgeons (Nederlandse Vereniging voor Heelkunde). The findings are expected to contribute to future updates of the Dutch proctology guideline. Several manuscripts arising from this research are expected to be submitted for publication in peer-reviewed journals, including publication of the study protocol itself.

### Monitoring and safety

Monitoring will be performed in compliance with Good Clinical Practice using a risk-based approach described in a separate monitoring plan. Monitoring activities will focus on informed consent, eligibility criteria, primary outcome verification and safety reporting. All adverse events reported spontaneously by participants or observed by investigators will be documented, and serious adverse events will be reported to the reviewing authority within mandated timelines.

A Data Safety Monitoring Board is not considered necessary because the trial compares two established treatment strategies that do not pose material incremental risk.

### Annual progress report and amendments

The sponsor/investigator will provide the review committee with an annual progress report. This report will include the date of inclusion of the first participant, the number of participants enrolled, the number who have completed the trial, any serious adverse events, other relevant problems and any amendments.

### Public disclosure and publication policy

All study results will be published without restrictions. All members of the study group agree that all study results will be published. Both negative and positive results will be published. Before publication, all authors will have the opportunity to give comments on the manuscript. Data will be published as soon as possible after finishing data analysis. The sponsor will have no influence on implementation of the research and content of publications. Publication of data will not take place until accrual of patients has been completed and after finishing data analysis.

The proposed study contributes to evidence-based medicine and the results should become available for routine daily practice. The implementation activities comprise general activities including dissemination activities and adoption of professional guidelines. The existing clinical guideline proctology will be adjusted: recommendations following from this study will be summarised in this guideline. The guideline is available to clinicians, patients, insurance companies and other healthcare providers. The result of implementation of the proposed study can be measured using the existing registration system. The DBC Information System (DIS) receives and manages information about all treatment processes in hospitals, mental healthcare and rehabilitation care. DIS is administratively and facility accommodated with the Dutch Healthcare Authority (DHA). Healthcare institutions have the legal obligation to provide the DHA with data on treatment. Implementation will further be propagated during annual society meetings of the Nederlandse Vereniging voor Heelkunde, NVVH (Dutch society of Surgery). The Nederlandse Vereniging voor Medische Microbiologie, NVMM, (Dutch society of Medical Microbiology) will also be contacted and members informed about our results. Resistance against results will be low due to high external validity (academic, top-clinic and general hospitals) which also facilitates implementation nationwide. To facilitate dissemination, education, and implementation, named investigators and an implementation expert are part of the research group.

### Data sharing statement

Data will be made available upon reasonable request. This will include deidentified participant data and statistical code. After an embargo period, the data may be accessed for further research and verification. Requests should be directed to the corresponding author and will be considered by the SETON trial study group.

## Supplementary material

10.1136/bmjopen-2026-122832 online supplemental file 1
