# Supplementary material for: Does omission of a loose seton before sphincter-preserving surgery achieve non-inferior fistula closure compared with routine loose seton placement in adults with single-tract cryptoglandular anal fistula in the Netherlands? Study protocol for the SETON multicentre randomised controlled non-inferiority trial and cost-utility analysis
Source: BMJ Open. 2026 Oct 6;16(10):e122832. doi: 10.1136/bmjopen-2026-122832 (PMC13646918; doi:10.1136/bmjopen-2026-122832)
Supplement: Supplementary file 1 [file bmjopen-2026-122832.reviewer_comments.pdf]

PEER REVIEW HISTORY

BMJ Open publishes all reviews undertaken for accepted manuscripts. Reviewers are asked to complete a checklist review form and are provided with free text boxes to elaborate on their assessment. These free text comments are reproduced below.

ARTICLE DETAILS

Title (Provisional)

Does omission of a loose seton before sphincter-preserving surgery achieve non-inferior fistula closure compared with routine loose seton placement in adults with single-tract cryptoglandular anal fistula in the Netherlands? Study protocol for the SETON multicentre randomised controlled non-inferiority trial and cost-utility analysis

Authors

Elwakel, Ahmed A; Van Hagen, Pieter; Veldkamp, Ruben; van Dieren, Susan; Bemelman, Willem A; Han-Geurts, Ingrid J.M.

This article was not externally reviewed at BMJ Open. Protocols that have been independently assessed prior to submission to BMJ Open are usually fast-tracked to publication on the grounds that further substantial changes will not be possible. This independent assessment will usually be external, independent review for both a substantial grant award from a non-commercial or government funder and ethics approval.
