# Supplementary material for: Does omission of a loose seton before sphincter-preserving surgery achieve non-inferior fistula closure compared with routine loose seton placement in adults with single-tract cryptoglandular anal fistula in the Netherlands? Study protocol for the SETON multicentre randomised controlled non-inferiority trial and cost-utility analysis
Source: BMJ Open. 2026 Oct 6;16(10):e122832. doi: 10.1136/bmjopen-2026-122832 (PMC13646918; doi:10.1136/bmjopen-2026-122832)
Supplement: Supplementary file 2 [file bmjopen-16-10-s001.docx]

**Proefpersoneninformatie voor deelname
aan medisch-wetenschappelijk onderzoek**

**De SETON trial: Seton plaatsen vóór de operatie of direct opereren bij een anale fistel: wat is beter?**

*Officiële titel: Losse seton vóór de operatie bij niet complexe cryptoglandulaire fistel met een enkelvoudige fistelgang versus geen seton: gerandomsieerde trial*

**Inleiding**

Geachte heer/mevrouw,

Met deze informatiebrief willen we u vragen of u wilt meedoen aan medisch-wetenschappelijk onderzoek. Meedoen is vrijwillig. U krijgt deze brief omdat de arts bij u een anale fistel heeft vastgesteld en u binnenkort een operatie moet ondergaan. Als u bekend bent met ontstekingsziekten van de darm of de ziekte van Crohn dan mag u niet meedoen aan dit onderzoek.
U leest hier om wat voor onderzoek het gaat, wat het voor u betekent, en wat de voordelen en nadelen zijn. Het is veel informatie. Wilt u de informatie doorlezen en beslissen of u wilt meedoen? Als u wilt meedoen, kunt u het formulier invullen dat u vindt in bijlage C.

**Stel uw vragen**

U kunt uw beslissing nemen met de informatie die u in deze informatiebrief vindt. Daarnaast raden we u aan om dit te doen:

- Stel vragen aan de onderzoeker die u deze informatie geeft.

- Praat met uw partner, familie of vrienden over dit onderzoek.

- Stel vragen aan de onafhankelijk deskundige. Voor contactgegevens zie bijlage A

- Lees de informatie op [www.rijksoverheid.nl/mensenonderzoek](http://www.rijksoverheid.nl/mensenonderzoek).

1. **Algemene informatie**

Dit onderzoek wordt uitgevoerd vanuit het Amsterdam UMC en de Proctos Kliniek (opdrachtgever), in samenwerking met meerdere Nederlandse ziekenhuizen. Dit onderzoek wordt gesubsidieerd door ZonMw, een Nederlandse organisatie voor gezondheidsonderzoek en zorginnovatie. De medisch-ethische toetsingscommissie Amsterdam UMC heeft dit onderzoek goedgekeurd. We verwachten ongeveer 277 deelnemers nodig te hebben.

Algemene informatie over de toetsing van onderzoek vindt u in de brochure ‘Medisch-wetenschappelijk onderzoek’.

**2. Wat is het doel van het onderzoek?**

We willen nagaan of een directe operatie zonder seton (een dun draadje dat 6-8 weken in de fistel wordt gebracht) minstens zo effectief is als de gebruikelijke behandeling (eerst seton plaatsing, gevolgd door operatie) bij fistelgenezing na 26 weken en hoe dit de patiëntbeleving en kosten beïnvloedt.

1. **Wat is de achtergrond van het onderzoek?**

Een anale fistel is een niet normale verbinding tussen endeldarm en huid. Dit is een veelvoorkomende aandoening die gepaard gaat met klachten van pijn, verlies van bloed, pus, ontlastingsresten en een aangetaste huid. Omdat genezing soms moeilijk verloopt, is het gebruikelijk eerst een seton (dun draadje dat tijdelijk in de fistel wordt gebracht) te plaatsen om afvoer te verbeteren en ontsteking te voorkomen. Het plaatsen van de seton gebeurt tijdens een korte ingreep onder verdoving (lokaal of algeheel), en de seton blijft meestal 6-8 weken zitten. Hoewel het in de huidige chirurgische praktijk gebruikelijk is om standaard een seton te plaatsen, is nog niet duidelijk of deze tussenstap echt nodig is. Daarom onderzoeken we wat de resultaten zijn wanneer patiënten direct aan hun fistel worden geopereerd. Dit kan mogelijk betekenen dat patiënten minder vaak geopereerd hoeven te worden en dat de kosten voor de gezondheidszorg worden verminderd.

2. **Hoe verloopt het onderzoek?**

Hoelang duurt het onderzoek?

Doet u mee met het onderzoek? Dan duurt dit voor u in totaal ongeveer 26 weken (6 maanden), met een aantal vaste meetmomenten.

De behandeling

Als u besluit om mee te doen aan dit onderzoek, wordt u via loting (randomisatie) ingedeeld in één van twee behandelgroepen:

•	In de eerste groep wordt bij u tijdens de eerste ingreep een zogenoemde seton geplaatst, gevolgd door de operatietechniek die samen met u gekozen is om de fistel te sluiten.

•	In de tweede groep wordt géén seton geplaatst, maar wordt de fistel direct chirurgisch behandeld met de operatietechniek die samen met u gekozen is om de fistel te sluiten.

Vragenlijsten

Wij vragen u om gedurende het onderzoek op een aantal momenten digitale vragenlijsten op de computer/tablet/laptop in te vullen. Dit gebeurt op vijf vaste momenten: vóór de operatie, 1 week, 6 weken, 6 maanden en 12 maanden na de operatie. Het invullen van een vragenlijst duurt ongeveer 10 minuten (zie bijlage C).

De vragenlijsten worden via een beveiligde online omgeving verstuurd. Hiervoor vragen wij uw toestemming om uw e-mailadres te gebruiken, zodat u de link naar de vragenlijsten digitaal kunt ontvangen. Zie bijlage C voor een overzicht van alle vragenlijsten en bezoeken.

Bezoeken en metingen

Zoals bij alle patiënten na de operatie, komt u ook na 6 maanden terug voor een controle op de polikliniek.. Tijdens deze controle kijkt de arts of de fistel goed geneest en of er nog klachten zijn. Indien nodig kan aanvullend onderzoek zoals een echografie of MRI worden verricht, bijvoorbeeld bij twijfel over de genezing of bij terugkeer van klachten.

3. **Welke afspraken maken we met u?**

U wordt gevraagd om:

- De toegewezen behandeling te ondergaan.
- Deel te nemen aan geplande controles.
- De vragenlijsten op de verschillende momenten invult.

Het is belangrijk dat u contact opneemt met de onderzoeker:

- Als u in een ziekenhuis wordt opgenomen of wordt behandeld voor dezelfde of een andere ziekte.
- Als u plotseling gezondheidsklachten krijgt.
- Als u niet meer wilt meedoen aan het onderzoek
- Als uw contactgegevens wijzigen.

4. **Van welke bijwerkingen, nadelige effecten of ongemakken kunt u last krijgen?**

Zowel bij de setonplaatsing als de operatie kunt u last krijgen van pijn, infecties, terugkeer van de fistel of tijdelijk verlies van ontlasting. De seton kan plaatselijk irritatie of ongemak geven. Dit zijn bekende risico’s van behandelingen, die niet verhoogd worden door deelname aan het onderzoek.

5. **Wat zijn de voordelen en de nadelen als u meedoet aan het onderzoek?**

**Mogelijke voordelen (zonder seton vóór de operatie)**

- U heeft één ingreep minder: er hoeft geen seton geplaatst of later verwijderd te worden.
- U vermijdt mogelijke seton-gerelateerde klachten, zoals lokale irritatie, pijn, jeuk, hygiëneproblemen of tussentijdse poliklinische controles/vervangingen.
- Mogelijk een kortere behandelroute met minder ziekenhuisbezoeken.

**Mogelijke nadelen (zonder seton vóór de operatie)**

- Een seton zorgt bij sommige mensen voor afvloed en “rust” in het fistelkanaal; zonder seton kunt u tot aan de operatie meer last houden van lekkage, viezigheid, jeuk of pijn.
- Bij een deel van de patiënten helpt een seton om de ontsteking te temperen; zonder seton kan de klachtenfase subjectief wat langer aanvoelen.

**Belangrijk om te weten**
Op dit moment is niet bewezen welke aanpak beter is: eerst een seton plaatsen of direct opereren zonder seton. Daarom doen we dit onderzoek. Door deelname helpt u mee om dit goed te vergelijken.

Wilt u niet meedoen?
U beslist zelf of u meedoet aan het onderzoek. Deelname is volledig vrijwillig. Als u niet wilt meedoen, krijgt u de gebruikelijke behandeling zoals deze in uw ziekenhuis wordt toegepast. Uw arts beslist dan samen met u of er een seton geplaatst wordt of niet, afhankelijk van uw situatie.

Komt er tijdens het onderzoek nieuwe informatie beschikbaar die belangrijk voor u is? Dan zal de onderzoeker u daarover informeren, en vragen of u wilt blijven deelnemen.

6. **Wanneer stopt het onderzoek?**

De onderzoeker laat het u weten als er nieuwe informatie over het onderzoek komt die belangrijk voor u is. De onderzoeker vraagt u daarna of u blijft meedoen.

In deze situaties stopt voor u het onderzoek:

- Het herhaal bezoek en de vragenlijst volgens het schema voorbij zijn.
- U kiest zelf om te stoppen
- Het hele onderzoek is afgelopen
- De onderzoeker het beter voor u vindt om te stoppen
- De Proctos Kliniek, de overheid of de beoordelende Medische-ethische toetsingscommissie Amsterdam UMC besluit om het onderzoek te stoppen.

Het hele onderzoek is afgelopen als alle deelnemers klaar zijn.

7. **Wat gebeurt er na het onderzoek?**

U ontvangt een overzicht van de belangrijkste resultaten zodra beschikbare uitkomsten zijn geanalyseerd, meestal binnen 18–24 maanden na het einde van uw deelname.

8. **Wat doen we met uw gegevens?**

Doet u mee met het onderzoek? Dan geeft u ook toestemming om uw gegevens te verzamelen, gebruiken en bewaren.

Welke gegevens bewaren we?

We bewaren deze gegevens:

- uw naam

- uw geslacht

- uw adres

- uw geboortedatum

- gegevens over uw gezondheid

- (medische) gegevens die we tijdens het onderzoek verzamelen

De vragenlijsten zullen door de projectgroep per mail worden verstuurd, hiervoor zullen zij uw e-mailadres ontvangen.

Waarom verzamelen, gebruiken en bewaren we uw gegevens?

We verzamelen, gebruiken en bewaren uw gegevens om de vragen van dit onderzoek te kunnen beantwoorden. En om de resultaten te kunnen publiceren. Gegevens kunnen worden gebruikt door de opdrachtgever en bedrijven die de opdrachtgever helpen bij het uitvoeren van de studie en het analyseren van onderzoeksgegevens

Hoe beschermen we uw privacy?

Om uw privacy te beschermen geven wij uw gegevens een code. Op al uw gegevens zetten we alleen deze code. De sleutel van de code bewaren we op een beveiligde plek in het ziekenhuis. Als we uw gegevens en lichaamsmateriaal verwerken, gebruiken we steeds alleen die code. Ook in rapporten en publicaties over het onderzoek kan niemand terughalen dat het over u ging.

Wie kunnen uw gegevens zien?

Sommige personen kunnen wel uw naam en andere persoonlijke gegevens zonder code inzien. Dit kunnen gegevens zijn die speciaal voor dit onderzoek zijn verzameld, maar ook gegevens uit uw medisch dossier.

Dit zijn mensen die controleren of de onderzoekers het onderzoek goed en betrouwbaar uitvoeren. Deze personen kunnen bij uw gegevens komen:

- Een controleur die door de opdrachtgever is ingehuurd.
- Nationale en toezichthoudende autoriteiten, bijvoorbeeld, de Inspectie Gezondheidszorg en Jeugd

Deze personen houden uw gegevens geheim. Voor inzage door deze personen vragen wij u toestemming te geven. De Inspectie Gezondheidszorg en Jeugd kan zonder uw toestemming uw gegevens inzien.

Hoelang bewaren we uw gegevens?

Uw gegevens worden 15 jaar bewaard op de onderzoekslocatie.

Mogen we uw gegevens gebruiken voor ander onderzoek?

Uw verzamelde gegevens kunnen ook van belang zijn voor ander wetenschappelijk onderzoek op het gebied van anale fistels . Daarvoor zullen uw 15 jaar worden bewaard op de onderzoekslocatie. In het toestemmingformulier geeft u aan of u dit goed vindt. Geeft u geen toestemming? Dan kunt u nog steeds meedoen met dit onderzoek. U krijgt dezelfde zorg.

Wat gebeurt er bij onverwachte ontdekkingen?

Tijdens een MRI-scan of echo kunnen we toevallig iets vinden dat niet direct van belang is voor het onderzoek maar wel voor uw gezondheid. De onderzoeker neemt dan contact op met uw huisarts of specialist. U bespreekt dan met uw huisarts of specialist wat er moet gebeuren. De kosten hiervan vallen onder uw eigen zorgverzekering. U geeft met het formulier toestemming voor het informeren van uw huisarts of specialist.

Kunt u uw toestemming voor het gebruik van uw gegevens weer intrekken?

U kunt uw toestemming voor het gebruik van uw gegevens op ieder moment intrekken. Zeg dat dan tegen de onderzoeker. Maar let op: trekt u uw toestemming in, en hebben onderzoekers dan al gegevens verzameld voor een onderzoek? Dan mogen zij deze gegevens nog wel gebruiken.

Wilt u meer weten over uw privacy?

- Wilt u meer weten over uw rechten bij de verwerking van persoonsgegevens? Kijk dan op www.autoriteitpersoonsgegevens.nl.
- Heeft u vragen over uw rechten? Of heeft u een klacht over de verwerking van uw persoonsgegevens? Neem dan contact op met het onderzoeksteam. Zie bijlage A.

Waar vindt u meer informatie over het onderzoek?

Op de volgende website(s) vindt u meer informatie over het onderzoek. www.onderzoekmetmensen.nl, [www.ClinicalTrials.gov](about:blank) en/of https://euclinicaltrials.eu.
Na het onderzoek kan de website een samenvatting van de resultaten van dit onderzoek tonen. U vindt het onderzoek door te zoeken op ‘SETON’

9. **Krijgt u een vergoeding als u meedoet aan het onderzoek?**

U krijgt geen vergoeding als u meedoet aan dit onderzoek.

10. **Bent u verzekerd tijdens het onderzoek?**

Voor iedereen die meedoet aan dit onderzoek is een verzekering afgesloten. De verzekering dekt schade door het onderzoek. Niet alle schade is gedekt. In bijlage B vindt u meer informatie over de verzekering en de uitzonderingen. Daar staat ook aan wie u schade kunt melden.

11. **We informeren uw huisarts**

Wij sturen uw huisarts altijd een brief om te laten weten dat u meedoet aan het onderzoek. Dit is voor uw eigen veiligheid. Als u dit niet goed vindt, kunt u niet meedoen aan dit onderzoek. U kunt niet deelnemen aan het onderzoek als u geen huisarts heeft.

**14. Patiënten vereniging**

Mocht u behoefte hebben aan

lotgenoten contact dan kunt u via de patiëntenorganisatie stichting Bekkenbodem4All (https://bekkenbodem4all.nl) daarmee in contact komen.

**15. Heeft u vragen?**

Indien u nog vragen heeft na het lezen van deze brief kunt u contact opnemen met de onderzoeker. Contactgegevens kunt u vinden in bijlage A.

Wanneer u behoefte heeft om over het onderzoek te praten met een arts die niet bij

het onderzoek betrokken is, maar die er wel van op de hoogte is, dan kunt u contact

opnemen met……

.

Buiten kantooruren kunt u voor spoedgevallen bellen met het algemene nummer van

uw ziekenhuis en vragen naar de dienstdoende chirurg.

Als u niet tevreden bent over het onderzoek of de behandeling kunt u terecht bij de

afdeling Patiëntenvoorlichting van uw ziekenhuis. De contactgegevens vindt u in

bijlage A.

**16. Hoe geeft u toestemming voor het onderzoek?**

Wanneer u voldoende bedenktijd heeft gehad, wordt u gevraagd te beslissen over

deelname aan dit onderzoek. Indien u toestemming geeft, zullen wij u vragen deze op

de bijbehorende toestemmingsverklaring schriftelijk te bevestigen. Door uw

schriftelijke toestemming geeft u aan dat u de informatie heeft begrepen en instemt

met deelname aan het onderzoek.

Eén exemplaar wordt door de onderzoeker bewaard. U krijgt een kopie of een tweede

exemplaar van deze brief met toestemmingsverklaring mee.

Dank voor uw tijd.

Met vriendelijke groet,

Dr. I.J.M. Han-Geurts, chirurg Proctos Kliniek te Bilthoven

**17. Bijlagen bij deze informatie**

A.	Contactgegevens

B.	Informatie over de verzekering

C.	Schema onderzoekshandelingen / omschrijving onderzoekshandelingen of overzicht metingen

D. Toestemmingsformulier

**Bijlage A: contactgegevens voor (per centrum in te vullen) .**

Hoofdonderzoeker:

(per centrum in te vullen)

Arts-onderzoeker:

Ahmed Elwakel

Onafhankelijke arts (chirurg) Amsterdam-UMC

Klachten

Indien u niet tevreden bent over het onderzoek of de behandeling kunt u terecht bij

de onafhankelijke klachtencommissie van (per centrum in te vullen) .

Naam: (per centrum in te vullen)	E: (per centrum in te vullen)

Functionaris voor de Gegevensbescherming van de instelling:

Per centrum in te vullen	E: (per centrum in te vullen)

**Bijlage B: informatie over de verzekering**

Voor iedereen die meedoet aan dit onderzoek, heeft de Proctos Kliniek een

verzekering afgesloten. De verzekering dekt schade door deelname aan het

onderzoek. Dit geldt voor schade tijdens het onderzoek of binnen vier jaar na het

einde van uw deelname aan het onderzoek. Schade moet u binnen die vier jaar aan

de verzekeraar hebben gemeld.

De verzekering dekt niet alle schade. Onderaan deze tekst staat in het kort welke

schade niet wordt gedekt.

Deze bepalingen staan in het 'Besluit verplichte verzekering bij medischwetenschappelijk onderzoek met mensen 2015'. Dit besluit staat in de Wettenbank van de overheid (<https://wetten.overheid.nl>).

Bij schade kunt u direct contact leggen met de verzekeraar.

De verzekeraar van het onderzoek is:

CNA Insurance Company (Europe) S.A.

35F, avenue John F. Kennedy, L-1855 Luxembourg

De verzekering biedt een dekking van € 650.000,00 per proefpersoon en € 5.000.000,00 voor het hele onderzoek en € 7.500.000,00 per jaar voor alle onderzoeken van dezelfde opdrachtgever.

De verzekering dekt de volgende schade niet:

- schade door een risico waarover u in de schriftelijke informatie bent ingelicht. Dit geldt niet als het risico zich ernstiger voordoet dan was voorzien of als het risico heel onwaarschijnlijk was;
- schade aan uw gezondheid die ook zou zijn ontstaan als u niet aan het onderzoek had meegedaan;
- schade door het niet (volledig) opvolgen van aanwijzingen of instructies;
- schade aan uw nakomelingen, als gevolg van een negatief effect van het onderzoek op u of uw nakomelingen;
- schade door een bestaande behandelmethode bij onderzoek naar bestaande behandelmethoden.

**Bijlage C: Overzicht van het onderzoekstraject**

Hieronder vindt u een eenvoudig overzicht van wat er van u wordt verwacht tijdens het onderzoek.

**Overzicht onderzoekshandelingen**

| **Moment** | **Wat gebeurt er?** |
| --- | --- |
| **Tijdens de operatie** | U wordt willekeurig ingedeeld in één van de twee behandelgroepen:  1. Met plaatsing van een losse seton vóór de definitieve operatie. 2. Zonder plaatsing van een seton vóór de definitieve operatie. U krijgt daarna de operatieve behandeling die door uw chirurg als passend wordt beoordeeld. |
| **Voor de operatie (baseline)** | Eerste vragenlijst over uw gezondheid en klachten. Circa 5 minuten |
| **Na 1 weken** | Online vragenlijst over uw herstel, pijn en kwaliteit van leven.  Circa 10 minuten |
| **Na 6 weken** | Online vragenlijst.  Circa 10 minuten |
| **Na 6 maanden (26 weken)** | Belangrijk meetmoment: – Online vragenlijst Circa 10 minuten – Fysieke controle op de polikliniek om te kijken of de fistel is genezen. |
| **Na 12 maanden** | Laatste online vragenlijst over uw gezondheidstoestand op langere termijn. Circa 10 minuten |

**Bijlage D: toestemmingsformulier proefpersoon**

**De SETON trial: setonplaatsing voorafgaand aan chirurgie vergeleken met directe chirurgische behandeling van een anale fistel**

- Ik heb de informatiebrief gelezen. Ook kon ik vragen stellen. Mijn vragen zijn goed genoeg beantwoord. Ik had genoeg tijd om te beslissen of ik meedoe.
- Ik weet dat meedoen vrijwillig is. Ook weet ik dat ik op ieder moment kan beslissen om toch niet mee te doen met het onderzoek. Of om ermee te stoppen. Ik hoef dan niet te zeggen waarom ik wil stoppen.
- Ik geef de onderzoeker toestemming om mijn huisarts en/of specialist te laten weten dat ik meedoe aan dit onderzoek.
- Ik geef de onderzoeker toestemming om mijn huisarts of specialist informatie te geven over onverwachte bevindingen uit het onderzoek die van belang zijn voor mijn gezondheid.
- Ik geef de onderzoekers toestemming om mijn gegevens te verzamelen en gebruiken. De onderzoekers doen dit alleen om de onderzoeksvraag van dit onderzoek te beantwoorden.
- Ik weet dat voor de controle van het onderzoek sommige mensen al mijn gegevens kunnen inzien. Die mensen staan in deze informatiebrief. Ik geef deze mensen toestemming om mijn gegevens in te zien voor deze controle.
- Ik geef toestemming voor het feit dat mijn emailadres inzichtelijk is voor de onderzoeker in het coördinerend centrum (Proctos Kliniek) voor het versturen van vragenlijsten.
- Wilt u in de tabel hieronder ja of nee aankruisen?

| Ik geef toestemming om mijn gegevens te bewaren om dit te gebruiken voor ander onderzoek, zoals in de informatiebrief staat. | Ja ☐ | Nee☐ |
| --- | --- | --- |
| Ik geef toestemming om mij eventueel na dit onderzoek te vragen of ik wil meedoen met een vervolgonderzoek. | Ja ☐ | Nee☐ |

- Ik wil meedoen aan dit onderzoek.

Mijn naam is (proefpersoon): ………………………………..

Handtekening: ………………………	Datum	: __ / __ / __

-----------------------------------------------------------------------------------------------------------------

Ik verklaar dat ik deze proefpersoon volledig heb geïnformeerd over het genoemde onderzoek.

Wordt er tijdens het onderzoek informatie bekend die die de toestemming van de proefpersoon kan beïnvloeden? Dan laat ik dit op tijd weten aan deze proefpersoon.

Naam onderzoeker (of diens vertegenwoordiger):……………………………….

Handtekening:………………………	Datum: __ / __ / __

-----------------------------------------------------------------------------------------------------------------

*De proefpersoon krijgt een volledige informatiebrief mee, samen met een getekende versie van het toestemmingsformulier.*
